# Correspondence

**Published:** 1846-03

**Authors:** A. Berry, C. P. Van Houten, C. Branham, Jas. Taylor

**Affiliations:** Committee of Class.; Committee of Class.; Committee of Class.


### ARTICLE VII.

### *Correspondence.*

*Cincinnati*, *Feb.* 4, 1846.

PROFESSOR HARRIS,

Dear Sir:—The class of the Ohio College of Dental Surgery, thinking, that a knowledge of the prospect of a work on Dental Pharmacy, from the pen of Prof. Taylor, might be interesting to many members of the profession, have instructed us to send the following correspondence for publication, if you think proper, in the Dental Journal.

Respectfully, yours,

A. BERRY,
C. P. VAN HOUTEN,
C. BRANHAM.

*Ohio College of Dental Surgery, Jan'y* 5, 1846.

PROFESSOR TAYLOR,

Dear Sir:—The members of your class having felt the want of a work on Dental Pharmacy, and being highly pleased with your excellent lectures on that subject, and believing that their publication, with those on the mode of making solder, and of refining and preparing gold, would supply an important desideratum in works on dental science, useful, not only to the student, but convenient for reference to the practitioner; they would, therefore, respectfully suggest to you the propriety of furnishing them for the press.

Please accept, in behalf of your class, our sentiments of respect and esteem. Yours, truly,

A. BERRY,

C. P. VAN HOUTEN, } *Committee of Class.*

C. BRANHAM,

---

*Cincinnati, Jan'y 6th,* 1846.

MESSRS. BERRY, VAN HOUTEN, and BRANHAM,

Gentlemen:—Your communication, as a committee of the dental class, has been duly received. It affords me unfeigned pleasure to know that any portion of my winter's labor has been acceptable to the class, and particularly that portion so kindly alluded to in your note.

No one can feel the want of such a work as that to which you allude, more than myself. It has, however, only the more convinced me of the fact, that pharmacy has been too much neglected by our profession.

My lectures, however, on this subject, were prepared without any reference to publication, and, in their present form, are not properly arranged for the press.

I look forward to the time when we shall have small practical works on all the different branches of dental science; when the library of the dental practitioner shall contain more than simply one or two works on practical dentistry.

A work embracing strictly that to which your note alludes, I should be glad to see from the hands of some one more capable of doing it justice than myself. I shall, however, if circumstances permit, during the coming summer, arrange my notes on this subject, that, after the next course of lectures in the Ohio College of Dental Surgery, (if their place should not be supplied,) they can be published.

Accept, gentlemen, for yourselves, and each member of the class you represent, my warm acknowledgments for the unremitting attention which has been paid, thus far, to my lectures.

Yours, very respectfully, &c.

JAS. TAYLOR.

